# *Fusobacterium nucleatum* in biopsied tissues from colorectal cancer patients and alcohol consumption in Korea

**DOI:** 10.1038/s41598-020-76467-7

**Published:** 2020-11-16

**Authors:** Myungsook Kim, Seung-Tae Lee, Songyi Choi, Hyukmin Lee, Sun Sung Kwon, Jung Hyun Byun, Young Ah Kim, Ki-Jong Rhee, Jong Rak Choi, Tae Il Kim, Kyungwon Lee

**Affiliations:** 1 Department of Laboratory Medicine and Research Institute of Bacterial Resistance, Yonsei University College of Medicine, 50-1 Yonsei-ro, Seodaemun-gu, Seoul, 03722 South Korea; 2 Department of Pharmacology, Yonsei University College of Medicine, Seoul, South Korea; 3 Department of Laboratory Medicine, Gyeonsang National University Hospital, Gyeongsang National University College of Medicine, Jinju, South Korea; 4 Department of Laboratory Medicine, National Health Insurance Service Ilsan Hospital, Goyang, South Korea; 5 Department of Biomedical Laboratory Science, Yonsei University College of Health Sciences, Wonju, South Korea; 6 Department of Internal Medicine, Yonsei University College of Medicine, 50-1 Yonsei-ro, Seodaemun-gu, Seoul, 03722 South Korea

**Keywords:** Clinical microbiology, Cancer

## Abstract

The roles of individual bacteria and their relationship in the development of colorectal cancer (CRC) remain unclear. We aimed to determine the prevalence of CRC-associated bacteria using quantitative real-time PCR (qPCR) or 16S rRNA analysis and the statistical correlations of patient demographics and clinical characteristics comprising alcohol consumption with CRC-associated bacteria. We determined the prevalence of five CRC-associated bacterial species in 38 CRC patients (39 samples) and 21 normal individuals using qPCR, and the relative abundance of bacterial taxa in the gut microbiome was assessed using 16S rRNA analysis. *Fusobacterium nucleatum* was the only bacterium that was significantly (*P* < 0.0001) more prevalent in the cancer tissue (82.1%) than in the normal tissue (0%) by qPCR. 16S rRNA analysis showed a significant correlation between six operational taxonomic units (OTUs), namely, the genera *Fusobacterium, Peptostreptococcus, Collinsella, Prevotella, Parvimonas*, and *Gemella,* in patients with CRC. An integrated analysis using 16S rRNA data and epidemiological characteristics showed that alcohol consumption was significantly correlated with the abundance of *Fusobacterium* OTUs. The correlation of alcohol consumption with the abundance of *Fusobacterium* OTUs in cancer tissue discovered using 16S rRNA analysis suggests a possible link between alcohol metabolism and subsequent tumorigenesis caused by *F. nucleatum.*

## Introduction

Colorectal cancer (CRC) is the third most common cancer type in men and the second most common in women worldwide, and it is the fourth leading cause of cancer-related deaths in the world^1^. The mechanisms underlying the development of CRC are not comprehensively understood; however, various risk factors are known to contribute to carcinogenesis in CRC, including age, presence of colon polyps, high consumption of red meat, obesity, smoking, and alcohol consumption^2^. Furthermore, the response to these risk factors seems to differ between ethnicities and geographical regions, which may affect the prevalence and prognosis of CRC.

Recently, the gut microbiota has been added to the list of CRC risk factors, as it has been implicated in the development of CRC and might contribute to CRC progression, as suggested by the dynamic “driver-passenger” model^3^. In the past few decades, it has been shown that among the diverse bacterial species that are a part of the gut microbiome, the presence of specific bacteria can play a role in colorectal carcinogenesis^4^. Bacteria-driven oncogenic mechanisms in CRC have been proposed to include the activation of Wnt signaling (enterotoxigenic *Bacteroides fragilis* (ETBF)^5^ and *Fusobacterium* species^6^), proinflammatory signaling (*Enterococcus faecalis*^7^ and *Streptococcus gallolyticus*^8^), and genotoxicity (colibactin-producing *Escherichia coli,* colB + *E. coli*^9^). These carcinogenic effects can occur from very early stages and over the multistep processes of CRC carcinogenesis and can be accompanied by shifts in the gut microbiome and metabolome^10^. Furthermore, a recent study showed that the gut microbiome may promote the progression of CRC^11^. Therefore, it is crucial to identify bacteria that may be associated with the development of CRC and to assess which other factors can elicit transformation from a healthy gut microbiome to a tumorigenic microenvironment.

Diet is the most well-known factor that influences the gut microbiome, and differences in diet can substantially affect the entire gut microenvironment^12^. In South Korea, CRC is one of the most prevalent types of cancer; however, few metagenomic studies have been conducted to determine the association between the gut microbiome and CRC development^13^. The composition of the gut microbiome may be influenced by factors other than diet, including alcohol consumption^14^. Numerous epidemiological studies have shown that alcohol consumption is strongly associated with the incidence of CRC^15^. Retrospective propensity score matching analysis (adjusted hazard ratio: 1.86) revealed that high alcohol consumption increased the development of CRC^16^. Alcohol consumption is considerably high in South Korea^17^ and may be a causative factor underlying the high prevalence of CRC in Korea^18^. However, to our knowledge, the effect of alcohol consumption on the gut microbiome has not been investigated thoroughly in South Korea.

In this study, we aimed to determine the prevalence of five CRC-associated bacteria (ETBF, *E. faecalis*, colB + *E. coli*, *F. nucleatum*, and *S. gallolyticus*) in the biopsied tissues of patients with CRC using quantitative real-time PCR (qPCR) and the relative abundance of operational taxonomic units (OTUs) by 16S rRNA analysis. Furthermore, we determined the associations between the epidemiological characteristics of CRC and the gut microbiome and alcohol consumption.

## Results

### Comparison of demographics and clinical characteristics of cases and controls

A total of 39 CRC samples and 21 control samples were examined (Table 1). No significant differences between the groups were observed with respect to epidemiological characteristics, apart from BMI and hypertension. BMI was lower in CRC patients (22.9 ± 2.9) than in controls (24.8 ± 2.7), and hypertension was more prevalent in controls (76.2%, n = 16/21) than in CRC patients (46.2%, n = 18/39). The CRC cases consisted of 71.8% colon cancers and 28.2% rectal cancers, with right- and left-sided cancers accounted for 28.2% and 71.8% of the cases, respectively.

**Table 1:** Comparison of demographics and clinical characteristics of CRC patients and controls.

Characteristic	Number (%) of patients		*P*-value
	Cases (n = 38)	Controls (n = 21)	
Age (years)	65.9 ± 10.8	63.6 ± 7.0	0.3440
Height (cm)	161.8 ± 8.6	160.7 ± 8.2	0.5200
Weight (kg)	60.1 ± 9.6	64.5 ± 11.5	0.1900
BMI	22.9 ± 2.9	24.8 ± 2.7	0.0150
Sex (F/M)	15/23	13/8	0.1120
Alcohol (drinker/nondrinker)	15/17	10/6	0.3680
Smoking (ever/never)	15/23	6/10	0.5330
Diabetes (yes/no)	31/8	5/2	0.6360
Hypertension (yes/no)	18/21	16/5	0.0310
**Indication for colonoscopy; n (%)**			
Screening	NA	21	
Colorectal cancer	38	NA	
**Histologic diagnosis; n (%)**			
Hyperplastic polyp	NA	1 (4.8)	
Tubular adenoma	NA	12 (57.1)^b^	
Adenocarcinoma; n (%)	39^a^	NA	
Stage			
Stage I	4 (10.3)	NA	
Stage II	9 (23.1)	NA	
Stage III	12 (30.8)	NA	
Stage IV	14 (35.9)	NA	
**Location of CRC; n (%)**			
Cecum	1 (2.6)	NA	
Ascending colon	5 (12.8)	NA	
Hepatic flexure	4 (10.3)	NA	
Transverse colon	1 (2.6)	NA	
Descending colon	3 (7.7)	NA	
Rectosigmoid junction	2 (5.1)	NA	
Sigmoid colon	12 (30.8)	NA	
Rectum	11 (28.2)	NA	

*NA* not applicable.

^a^One patient had two adenocarcinomas: one in the hepatic flexure and one in the rectum.

^b^Four of the 12 controls with tubular adenoma had their tubular adenomas removed during colonoscopy.

### Prevalence of CRC-associated bacteria as assessed by qPCR

The prevalence of the five CRC-associated bacteria was examined in the carcinoma tissues (CTs) of CRC patients and in normal tissues (NTs) of controls using qPCR (Table 2). *F. nucleatum* was most frequently detected in CRC patients and was not detected in the NTs of controls (82.1% and 0%, respectively; *P* < 0.0001). However, no significant difference in the prevalence of colB + *E. coli,* ETBF, *E. faecalis*, or *S. gallolyticus* was observed between CRC patients and controls.

**Table 2:** Prevalence of CRC-associated bacteria by quantitative real-time PCR (qPCR).

Bacteria	No. (%) of patients positive for qPCR		*P*-value^c^
	CRC patients (n = 39)^a^	Controls (n = 21)^b^	
*Fusobacterium nucleatum*	32 (82.1)	0 (0.0)	< 0.0001
colB + *E. coli*	15 (38.5)	4 (19.0)	0.1538
*bft* + *Bacteroides fragilis*	14 (35.9)	8 (38.1)	1.0000
*Enterococcus faecalis*	6 (15.4)	1 (4.8)	0.4037
*Streptococcus gallolyticus*	0 (0.0)	1 (4.8)	0.3500

^a^No. of positives based on detection in the carcinoma tissues of CRC patients.

^b^No. of positives based on detection in the left and/or right normal tissues of controls.

^c^P-values were calculated using Fisher’s exact test.

### Prevalence of *F. nucleatum* by tumor stage and tissue type

**Figure 1:**
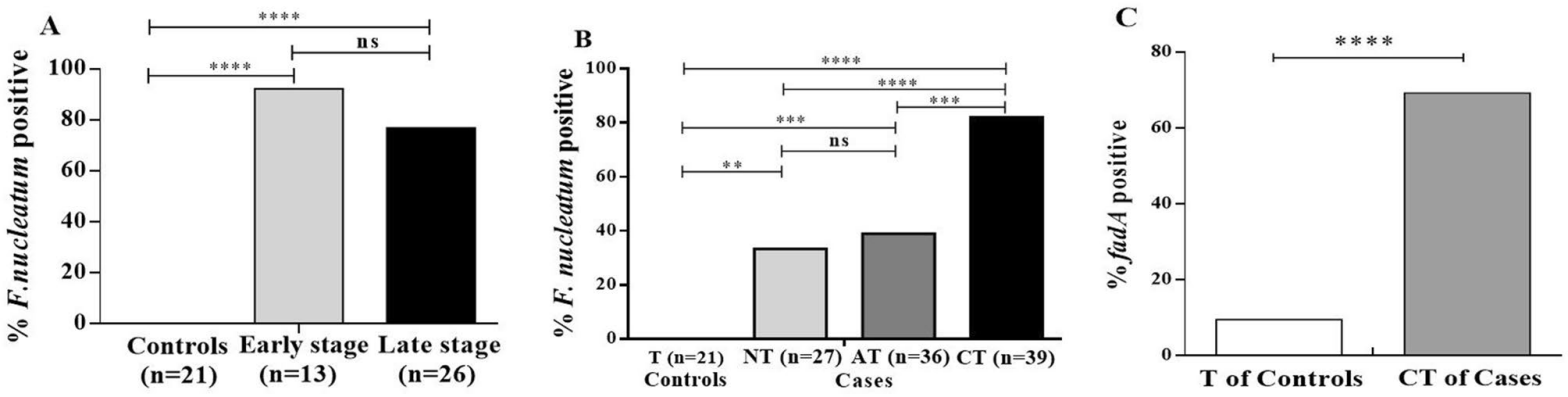
*F. nucleatum* statuses of CRC patients and controls. (**A**) Patients with CRC were categorized according to tumor stage (I/II: early stage, III/IV: late stage). *F. nucleatum* prevalence was significantly higher in CRC patients at early and late stages than in controls (92% and 76.9% vs. 0%, respectively; Fisher’s exact test, *P* < 0.0001, each). (**B**) Tissues of CRC patients were collected from carcinoma tissues (CTs), adjacent normal tissues (ATs) and/or normal tissues (NTs) from non-CRC sites. *F. nucleatum* was significantly higher in CTs (82.1%) than in ATs (38.9%) and NTs (33.3%) of CRC patients (CT vs. AT, CT vs. NT, AT vs. T, and NT vs. T; Fisher’s exact test, *P* = 0.0002, < 0.0001, 0.0008, and 0.003, respectively) and was significantly higher in CTs than in tissues (Ts) of controls (*P* < 0.0001). (**C**) Comparison of the prevalence of the *fadA* gene revealed significant differences between the CTs of patients and tissues (Ts) of controls (69.2% vs. 9.5%; Fisher’s exact test,* P* < 0.0001). Four asterisks (****) indicate *P* < 0.0001, three asterisks (***) indicate *P* < 0.001, and two asterisks (**) indicate *P* < 0.001; Fisher’s exact test was performed using GraphPad Prism version 5.0 for Windows.

We analyzed the association of *F. nucleatum* positivity in CRC patients with that in controls based on clinicopathological features. Patients with CRC were classified by tumor stage (early stage [I/II] or late stage [III/IV]). *F. nucleatum* was detected very frequently in both early (92.3%) and late (76.9%) stages; however, this difference was not significant (Fig. 1A). With respect to the tissue type, the prevalence of *F. nucleatum* was significantly higher in CTs (82.1%) than in adjacent normal tissues (ATs) (39.9%) as well as distal normal tissues (DTs) (33.3%) in CRC patients; no significant difference was observed between ATs and DTs (Fig. 1B). When the cycle threshold (Ct) values obtained from qPCR were compared among tissue types in *F. nucleatum*-positive cases, they were found to be significantly lower in CTs (Mann–Whitney U test, *P* < 0.0001). *F. nucleatum* was markedly enriched in CTs compared to ATs (Supplementary Fig. S1). The *fadA* gene, which encodes a virulence factor implicated in adhesion and invasion, was statistically more frequent in the CTs of CRC patients (69.2%) than in the NTs of controls (9.5%) (Fig. 1C).

### 16S rRNA analysis

Sequencing of the V3 and V4 regions in the 16S rRNA gene produced 171,988 ± 81,412 sequencing reads, on average. After quality filtration, clustering and taxonomy assignment were performed at the genus level by QIIME software^19^. A total of 698 OTUs were generated and used for further statistical analysis. In a diversity analysis, no significant difference in α- or β-diversity was observed between CRC cases and controls (Supplementary Fig. S2).

**Figure 2:**
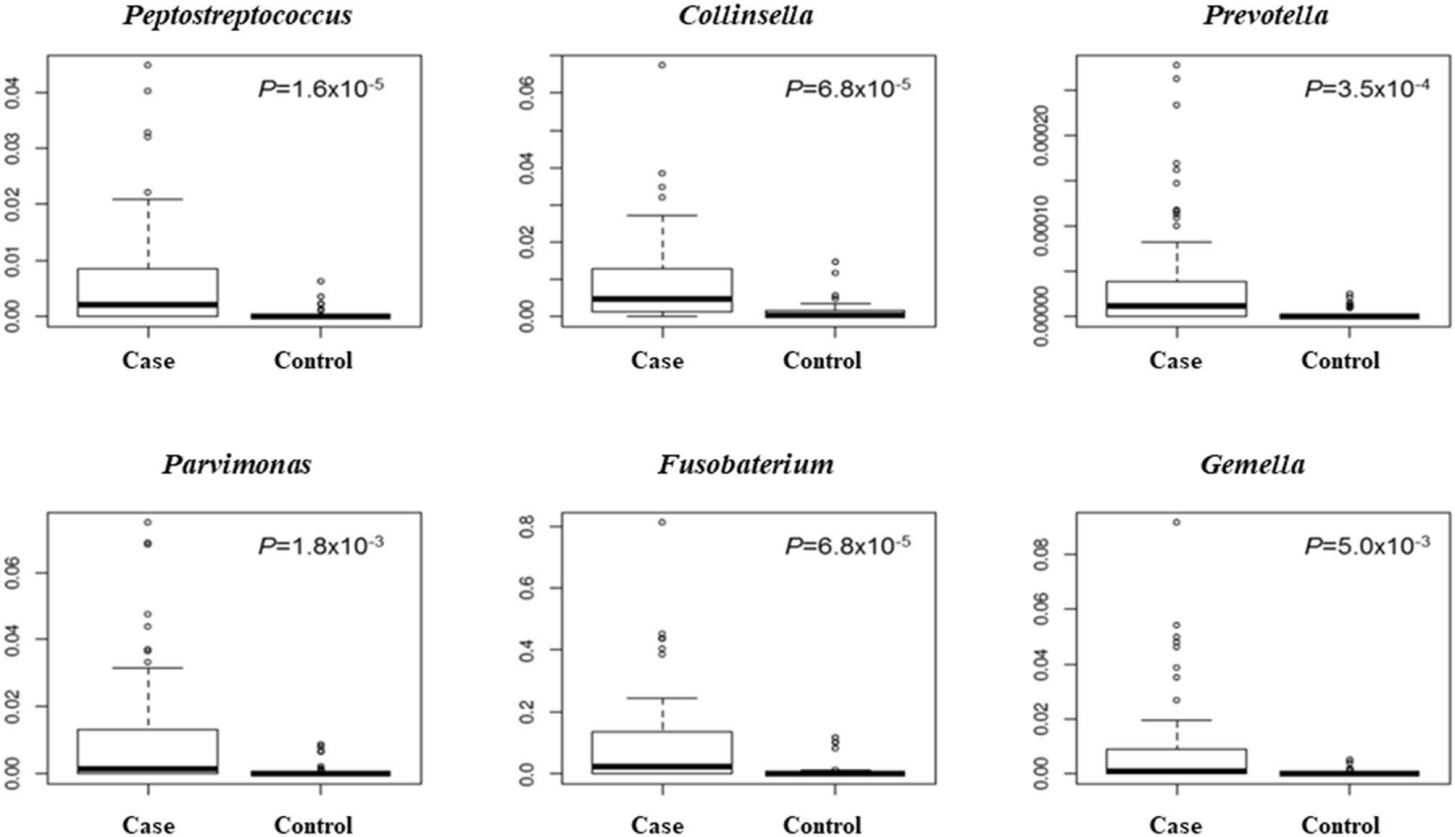
Relative abundance of six OTUs that were significantly different between cases (CRC patients) and controls were compared using R software.

**Figure 3:**
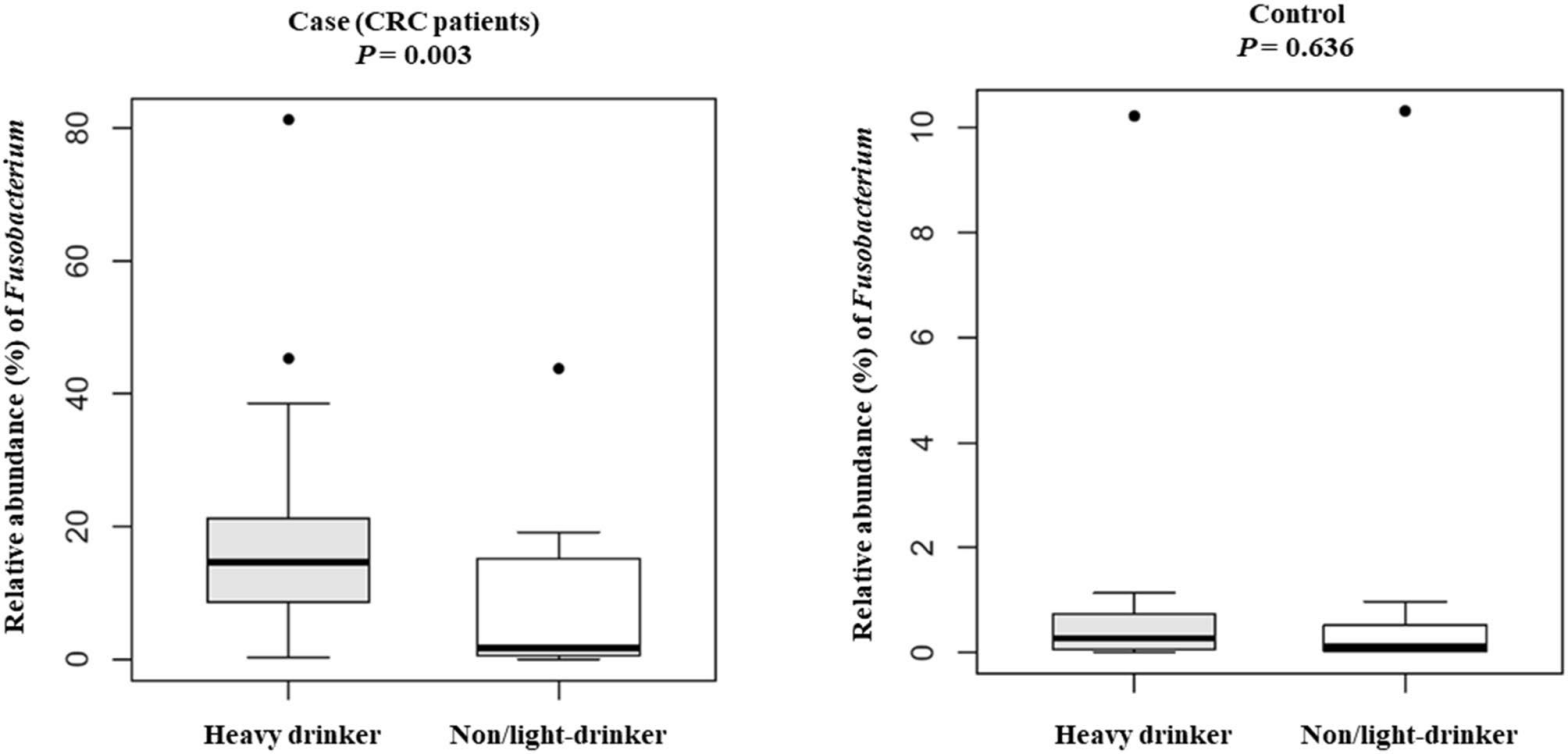
Differences in the proportion of *Fusobacterium* between heavy drinkers and non/light drinkers by cases (CRC patients) and controls. The proportion of *Fusobacterium* OTUs was significantly higher in heavy drinkers than in non/light drinkers (*P* = 0.003); no corresponding pattern was observed in controls. We used R software to perform the comparison and generate the figure.

**Figure 4:**
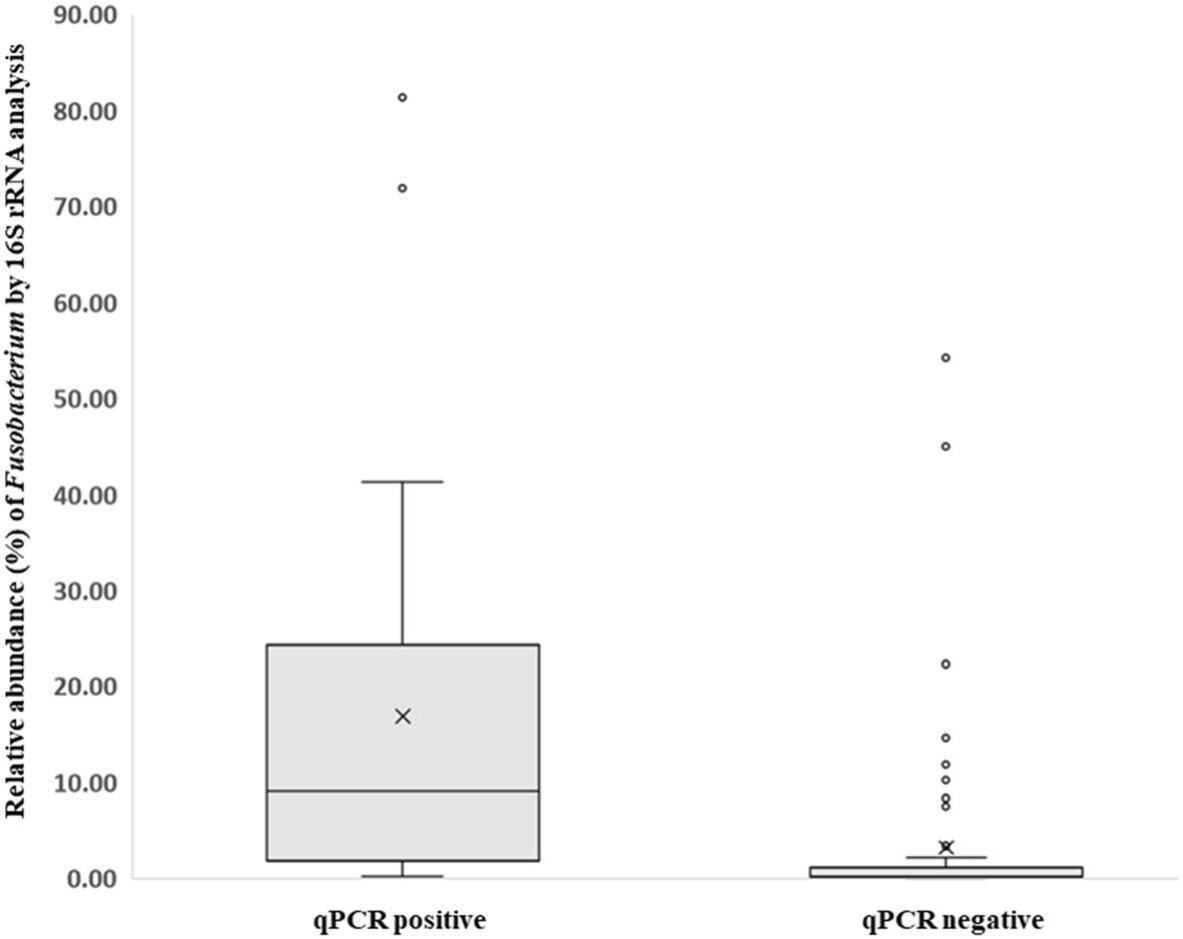
Box and whisker plots of the relative abundance of *Fusobacterium* by 16S rRNA analysis based on *F. nucleatum* qPCR positivity. Relative abundance (expressed as OTU percentages) was log-transformed for plotting on the Y-axis. Analysis was conducted with R software.

When comparing CRC cases with controls, the proportions of six OTUs, namely, *Peptostreptococcus, Collinsella, Prevotella, Parvimonas, Fusobacterium*, and *Gemella*, were significantly different after false discovery rate correction for multiple testing (Fig. 2). Among the epidemiological characteristics with continuous values, *Fusobacterium*-positive CRC patients were significantly younger than *Fusobacterium*-negative patients (64.0 ± 10.5 vs. 73.1 ± 9.3; *P* = 0.034), which was also true for *Parvimonas*-positive patients (60.4 ± 8.5 vs. 69.0 ± 10.9; *P* = 0.011; Table 3). With respect to epidemiological characteristics with binomial values, *Fusobacterium* was associated with alcohol consumption and *KRAS* mutation (*P* = 0.088 and *P* = 0.094), and *Parvimonas* was associated with tumor location and *KRAS* mutation, although the difference was only marginally significant (*P* = 0.070, Table 4). *Fusobacterium* was observed in 14 of the 15 heavy drinkers (93.3%) but not in 11 of the 17 non/light drinkers (64.7%). The proportion of *Fusobacterium* OTUs was significantly higher in heavy drinkers than in non/light drinkers (*P* = 0.003); no corresponding pattern was observed in controls (Fig. 3). With respect to tumor location, the proportion of the *Parvimonas* OTU was significantly higher in the descending colon and in the rectum (P < 0.001; Supplementary Fig. S3) than in other parts of the colon. Tumor stage and metastasis were not significantly correlated with the presence of specific bacteria. When the *Fusobacterium* OTU proportions were compared between cases and controls using Ct values, qPCR-positive cases showed higher OTU abundance values than qPCR-negative cases, and the significant positive correlation between these values is shown in Fig. 4.

**Table 3:** Differences in epidemiological characteristics with continuous values based on the association of CRC with the six OTUs.

Genus	Relative abundance ≥ 1%	Age	BMI	Tumor size	CEA	Total cholesterol
*Fusobacterium*	Positive	64.0 ± 10.5	22.7 ± 2.5	5.0 ± 2.4	236.8 ± 760.6	175.6 ± 43.9
	Negative	73.1 ± 9.3	23.6 ± 4.2	5.2 ± 1.9	234.6 ± 458.7	172.6 ± 54.2
	*P*-value	0.034*	0.609	0.883	0.992	0.890
*Parvimonas*	Positive	60.4 ± 8.5	22.4 ± 2.7	4.0 ± 1.8	512.5 ± 1118.3	182.8 ± 54.9
	Negative	69.0 ± 10.9	23.2 ± 3.0	5.3 ± 2.3	92.7 ± 268.9	170.9 ± 40.4
	*P*-value	0.011*	0.427	0.281	0.206	0.499
*Gemella*	Positive	63.2 ± 10.1	22.6 ± 2.4	4.4 ± 1.8	302.3 ± 540.4	173.2 ± 58.3
	Negative	67.4 ± 11.1	23.1 ± 3.2	5.3 ± 2.4	202.0 ± 781.5	175.8 ± 38.5
	*P*-value	0.240	0.606	0.403	0.646	0.886
*Prevotella*	Positive	65.4 ± 11.6	22.2 ± 1.9	5.3 ± 2.2	555.0 ± 1108.7	172.2 ± 45.4
	Negative	66.2 ± 10.6	23.3 ± 3.3	4.9 ± 2.4	70.6 ± 249.3	176.4 ± 46.3
	*P*-value	0.843	0.196	0.719	0.145	0.794
*Peptostreptococcus*	Positive	63.5 ± 11.9	23.0 ± 2.4	4.4 ± 1.4	524.9 ± 1177.6	169.6 ± 36.1
	Negative	66.8 ± 10.5	22.9 ± 3.1	5.4 ± 2.5	118.7 ± 344.8	177.1 ± 49.2
	*P*-value	0.434	0.896	0.283	0.285	0.609
*Collinsella*	Positive	65.8 ± 11.3	22.9 ± 3.0	NA	224.1 ± 714.0	171.9 ± 44.1
	Negative	66.8 ± 5.3	22.7 ± 1.4	NA	340.5 ± 677.6	200.8 ± 55.9
	*P*-value	0.780	0.774	NA	0.763	0.384

*NA* not applicable.

*Statistically significant at *P* < 0.05.

**Table 4:** Differences in epidemiological characteristics with binomial values based on the association of CRC with the six OTUs.

Genus	Relative abundance ≥ 1%	Sex		Diabetes		Smoking		Alcohol^a^		Hypertension		Tumor side		Metastasis		*KRAS* mutation		EGFR expression	
		M	F	Yes	No	Yes	No	Yes	No	Yes	No	Left	Right	Yes	No	Yes	No	Yes	No
*Fusobacterium*	Positive	18	12	7	23	13	17	14	11	15	15	6	24	11	18	9	7	8	5
	Negative	5	3	1	7	2	6	1	6	5	3	4	4	4	4	0	4	2	1
	*P*-value	0.999		0.660		0.440		0.088^†^		0.697		0.170		0.690		0.094^†^		0.999	
*Parvimonas*	Positive	9	5	3	11	7	7	7	3	8	6	1	13	5	8	1	6	3	3
	Negative	14	10	5	19	8	16	8	14	12	12	9	15	10	14	8	5	7	3
	*P*-value	0.999		0.999		0.492		0.128		0.745		0.059^†^		0.999		0.070^†^		0.607	
*Gemella*	Positive	8	5	2	11	6	7	8	5	8	5	2	11	6	6	2	5	4	3
	Negative	15	10	6	19	9	16	7	12	12	13	8	17	9	16	7	6	6	3
	*P*-value	0.999		0.689		0.728		0.280		0.506		0.441		0.488		0.374		0.999	
*Prevotella*	Positive	6	6	3	9	4	8	6	3	7	5	3	9	7	5	2	5	3	3
	Negative	17	9	5	21	11	15	9	14	13	13	7	19	8	17	7	6	7	3
	*P*-value	0.481		0.689		0.728		0.243		0.734		0.999		0.164		0.374		0.607	
*Peptostreptococcus*	Positive	7	4	2	9	5	6	5	4	6	5	2	9	5	6	1	4	4	1
	Negative	16	11	6	21	10	17	10	13	14	13	8	19	10	16	8	7	6	5
	*P*-value	0.999		0.999		0.722		0.699		0.999		0.690		0.728		0.319		0.588	
*Collinsella*	Positive	21	13	7	27	14	20	13	16	16	18	10	24	14	19	8	10	10	5
	Negative	2	2	1	3	1	3	2	1	4	0	0	4	1	3	1	1	0	1
	*P*-value	0.999		0.999		0.999		0.589		0.107		0.556		0.633		0.999		0.375	

^†^Marginally significant.

^a^Alcohol consumption; Yes, heavy drinker; No, non/light drinker.

## Discussion

CRC is a major health threat in many countries; however, its etiology and underlying mechanisms are still not comprehensively understood. The determination of a cancer’s etiology can result in the development of preventive or therapeutic measures. Several studies have demonstrated enrichment of fecal or tissue samples of CRC patients with specific bacterial pathogens.

ETBF is a well-known pathogen of the gastrointestinal tract that can trigger a carcinogenic multistep process through *B. fragilis* toxin^3–5,20^. However, we observed no significant difference in the prevalence of ETBF between CRC cases and controls, and its prevalence (35.9%) was significantly lower than that in a previous study (88.5%)^20^. In a recent study^21^, it was shown by qPCR that only 36.4% of CRC cases were positive for ETBF, and an equal distribution of *B. fragilis* was found in tumors, paired normal tissue and diverticula. Thus, the prevalence and distribution of ETBF should be determined by follow-up investigations. It has been shown that pathogenic *E. coli* can synthesize colibactin, which is genotoxic^22^. The prevalence of colB + *E. coli* in CRC patients might be the link between *E. coli* and CRC. In our study, the prevalence of colB + *E. coli* in CRC cases was twice as high as that in controls, but the difference was not statistically significant, probably due to an insufficient sample size. *S. gallolyticus* and *E. faecalis* were also regarded as CRC-associated bacteria in previous reports^23,24^; however, recent studies showed that the roles of *S. gallolyticus* and *E. faecalis* in CRC carcinogenesis were controversial due to their low prevalence^21,25,26^. In our study, neither species was frequently detected in cancer patients (Table 2).

In recent decades, *F. nucleatum* has attracted attention as a potential cause of CRC^4,6,27^. The role of *F. nucleatum* in CRC pathogenesis has not been comprehensively understood, but at least four mechanisms have been suggested to describe the same^28^: (1) cell proliferation through Wnt signaling by an interaction between FadA and E-cadherin, (2) antitumor immune evasion via Fap2 and T cell immunoreceptors having Ig and immunoreceptor tyrosine-based inhibitory motif domains, (3) tumor binding and enrichment of Fap2 and Gal-GalNAc, and (4) chemoresistance by lipopolysaccharide and Toll-like receptor 4.

*Fusobacterium* species are strictly anaerobic and are difficult to isolate by culture. In this study, the prevalence of *F. nucleatum* in the tissues of CRC patients as tested by qPCR (82.1%) was notably higher than that obtained using anaerobic culture (7.7%; data not shown). Therefore, non-culture-dependent detection methods such as qPCR or 16S rRNA analysis may be essential for screening *Fusobacterium* species or for studying its epidemiology in a population during the development of CRC.

The presence of *F. nucleatum* was significantly correlated with both the early and late stages of CRC (Fig. 1A). This finding suggests that *F. nucleatum* may be involved in CRC carcinogenesis from an early stage, and one review indicated an association of *F. nucleatum* with carcinomas throughout the different stages of CRC progression^29^. Analysis of the abundance of *F. nucleatum* by tissue type showed that the bacteria seemed to be more prevalent in CTs than in ATs and NTs (Fig. 1B). This finding is consistent with previous studies showing that a high level of *Fusobacterium* colonization is associated with CRC^27,30^.

FadA is a well-known oncogenic virulence factor that can induce oncogenic gene expression and promote the growth of CRC cells^6^. Therefore, FadA is regarded as a major virulence factor of *F. nucleatum* in CRC, and 69.2% of CT samples were positive for the *fadA* gene. However, some discrepancies between *F. nucleatum* presence and *fadA* gene frequency were observed in our study (Fig. 1C). Thus, it may be necessary to evaluate both *F. nucleatum* presence and *fadA* gene frequency to accurately determine the presence and virulence of *F. nucleatum*^31^. Interestingly, a recent study suggested that the *fadA* gene is not specific to *F. nucleatum* and that it is also present in other *Fusobacterium* species^32^. This finding indicates the need for further research regarding the role of other *Fusobacterium* species in CRC carcinogenesis.

The gut microbiome comprises numerous bacterial species that interact with each other, and it is possible that other bacteria in addition to the five known CRC-associated bacteria that were examined in the present study may affect the carcinogenesis of CRC. Thus, CRC metagenomic analysis is now commonly used to analyze this complex bacterial community. According to our 16S rRNA analysis, six genera, namely, *Peptostreptococcus, Collinsella, Prevotella, Parvimonas, Gemella*, and *Fusobacterium* were significantly abundant in CRC patients. Kwong et al. reported that *P. anaerobius* promoted CRC carcinogenesis in Apc^Min/+^ mouse models, and a correlation between *Peptostreptococcus* species and human CRC was reported previously^33^. The role of *Collinsella* species in CRC has rarely been investigated; however, Ai et al. reported that *Collinsella* species were specifically correlated with CRC in the Chinese population using metagenomic analysis^34^. The association of *Prevotella* species with CRC was reported in a previous study^35^, and our results are compatible with this report. *Parvimonas* and *Gemella* species were also reported as potential causative agents that may be responsible for CRC carcinogenesis^36^. However, it is difficult to determine which bacteria are the primary cause of CRC, as the gut microbiome is too complex and can vary by population^37^.

Based on an integrated analysis of 16S rRNA data and epidemiologic characteristics, we found a marginally significant correlation between *Fusobacterium* occurrence and a history of alcohol consumption. It has been shown that alcohol consumption is one of the major contributors to CRC carcinogenesis^38^. The generation of acetaldehyde and other metabolites from alcohol activates cancer-promoting signals^39^. Moreover, it was suggested that ethanol oxidation by intestinal anaerobes, including *Fusobacterium* species, under aerobic conditions in the colon and rectum may also play an important role in the pathogenesis of ethanol-related CRC^40^. To our knowledge, the present study was the first to determine the relationship between alcohol consumption and the presence of *Fusobacterium* using 16S rRNA analysis. In the integrated statistical analysis, a cutoff of 1% was used to determine the positivity of each OTU^41^. It is interesting to note that 87.5% (28/32) of qPCR-positive cases and 76.2% (16/21) of qPCR-negative controls showed the same 16S rRNA analysis results when a cutoff of 1% was used. This cutoff seems to be the appropriate threshold to determine abundance in 16S rRNA analysis in CRC tissues, though more investigations are required to confirm the cutoff.

The limitations of this study were the relatively small sample size, which was associated with low statistical significance, and the fact that tubular adenoma could not be analyzed as an independent group due to the small sample size. However, the simultaneous analysis of the presence of specific bacterial pathogens, 16S rRNA analysis, patient demographics and clinical characteristics may be an advantage of this study. The detection of *F. nucleatum* in stool can be helpful for diagnosing CRC in patients as a biological tumor marker if used concurrently with other tumor markers, and an appropriate testing method should be developed and validated.

In conclusion, *F. nucleatum* was significantly prevalent in the CTs of patients with CRC and frequently present in both early and late stages. These data support that this bacterium is strongly associated with the development of CRC. The correlation between alcohol consumption and the abundance of *Fusobacterium* OTUs in cancer tissue discovered using 16S rRNA analysis suggests a possible link between alcohol metabolism and changes in the gut microbiome and subsequent tumorigenesis caused by *F. nucleatum*.

## Methods

### Study population

A total of 38 CRC patients and 21 controls who were diagnosed using colonoscopy at a tertiary-care hospital between June 2015 and November 2016 were enrolled. Patients who received preoperative radiation, chemotherapy, and/or antibiotic treatment within 4 weeks were excluded. One patient had two primary adenocarcinoma lesions; thus, 39 CRC samples were analyzed in this study. Among the 21 controls, 12 individuals had tubular adenomas removed during colonoscopy, and 9 individuals showed no specific findings in colonoscopy. Patients with tubular adenomas were analyzed as controls because most of the polyps could be regarded as low risk due to their pathologic findings and small size (< 10 mm), and the resection margin was clear in all samples. G* power 3.1.9.7 analysis was conducted using the chi-square test to justify the appropriate sample size, and the test showed a significance level of 0.05 and a power of 0.81. Patient demographics and clinical characteristics were investigated by reviewing the medical records and interviews. Total alcohol consumption was calculated based on the history of alcohol intake—determined based on factors such as beverage type, amount, and frequency of consumption—and patients were classified as non/light drinkers (0‒1 drink/day) or heavy drinkers (≥ 2 drinks/day). All patients were classified as smokers (persons who are/were smokers) or nonsmokers (persons who never smoked) based on their smoking history.

### Ethics approval and consent to participate

This study was approved by the Institutional Review Board at Severance Hospital, Yonsei University, Republic of Korea (approval number: 2014-2768-004). All methods were performed following the relevant guidelines and regulations. Documented informed consent was obtained from each study participant.

### Colon tissue collection

Colonoscopy biopsies were obtained from the carcinoma tissues (CTs), adjacent normal tissues (ATs), and/or distant normal tissues (DTs) of CRC patients and from the right (from the cecum to transverse) and left (from descending to the rectum) colons of controls. Tissue samples were categorized as 'normal tissue' (NT) if no macroscopic or pathological evidence of carcinoma was observed. Tissue biopsies were collected in Transystem tubes (Copan, Brescia, Italy) containing anaerobic transport medium. Culture and DNA extraction for qPCR were performed within 30 min after tissue collection, and the remaining tissues were stored in a deep freezer (− 70 ℃) for 16S rRNA analysis. Detailed characteristics of CRC patients and controls are summarized in Supplementary Tables S1A,B.

### Detection of CRC-associated bacteria using qPCR

DNA was directly extracted from colon tissue using the QIAamp DNA Micro Kit (QIAGEN, Hilden, Germany) according to the manufacturer’s instructions. DNA concentration and purity were recorded using a NanoDrop spectrophotometer (NanoDrop Technologies, Montchanin, U.S.A.). The specific 16S ribosomal RNA (rRNA) genes were amplified by qPCR using the CFX96 Real-Time PCR System (Bio-Rad, Hercules, CA) to detect five CRC-associated bacteria: ETBF^42^, colB + *E. coli*^43^, *E. faecalis*^44^, *F. nucleatum*^27^, and *S. gallolyticus*^45^. The reaction mixture consisted of 10 µL of Ex Taq (TaKaRa, Kusatsu, Japan), 0.4 µL of each specific primer pair (10 µM), and 25 ng/µL DNA template in a total reaction volume of 20 µL. *E. faecalis* ATCC 29212, *F. nucleatum* subsp. *nucleatum* ATCC 25586 and three clinical isolates (ETBF, colB + *E. coli* and *S. gallolyticus*) were used for qPCR quality control. Identification of the clinical isolate was performed using MALDI-TOF mass spectrometry and 16S rRNA sequencing. For *S. gallolyticus*, the *rpoB* gene was also sequenced. The presence of the *fadA* gene in *F. nucleatum* was determined using the conventional PCR method in accordance with a previous study^31^, and the 211 bp size of the amplicon was confirmed by sequencing.

### 16S rRNA analysis

The V3 and V4 regions of the 16S rRNA gene (400–450 bp) were PCR amplified using target-specific primers and adapters for Illumina sequencing. Next-generation sequencing was performed on a MiSeq platform (Illumina, San Diego, USA) using the MiSeq Reagent Kit V2 with 500 cycles. Paired-end sequences were merged using PANDASEQ software^46^, and clustering of operational taxonomic units (OTUs) and taxonomy assignment were performed using QIIME software^19^. The observed diversity and Chao1 and Shannon indexes for α-diversity were calculated using QIIME. Principal components were calculated and plotted for β-diversity estimation. The relative proportion of each OTU was compared between cases and controls as well as with respect to epidemiological characteristics. OTUs present in > 1% of all OTUs were regarded as positive^16^. The mock community was analyzed before clinical specimen testing to validate the 16S rRNA analysis results.

### Statistical analyses

For 16S rRNA analysis, correction for multiple testing was performed using the false discovery rate (FDR) method. All statistical analyses were performed using GraphPad Prism version 5.0 (https://www.graphpad.com/scientific-software/prism/, GraphPad Software, La Jolla, CA, USA; Tables 1, 2, Fig. 1) and R software (https://www.R-project.org, The R Foundation, version 3.5.3; Tables 3, 4, Figs. 2, 3, 4)^47^. A value of P < 0.05 was defined as statistically significant, and a P-value between 0.05 and 0.10 was considered marginally significant.

## Supplementary information

Supplementary Information.

## Data Availability

The datasets generated and/or analyzed during the current study are available from the corresponding author upon reasonable request**.**
