# Supplementary material for: Fusobacterium nucleatum in biopsied tissues from colorectal cancer patients and alcohol consumption in Korea
Source: Sci Rep. 2020 Nov 16;10:19915. doi: 10.1038/s41598-020-76467-7 (PMC7669878; doi:10.1038/s41598-020-76467-7)
Supplement: Supplementary file 1 — Supplementary Information. [file 41598_2020_76467_MOESM1_ESM.docx]

Title: *Fusobacterium nucleatum* in biopsied tissues from colorectal cancer patients and alcohol consumption in Korea

Author: Myungsook Kim^1^, Seung-Tae Lee^1^, Songyi Choi^1^, Hyukmin Lee^1^, Sun Sung Kwon^2^, Jung Hyun Byun^3^, Young Ah Kim^4^, Ki-Jong Rhee^5^, Jong Rak Choi^1^, Tae Il Kim^6*^, Kyungwon Lee^1*^,

Affiliations

1. Department of Laboratory Medicine and Research Institute of Bacterial Resistance, Yonsei University College of Medicine, Seoul, South Korea
2. Department of Pharmacology, Yonsei University College of Medicine, Seoul, South Korea
3. Department of Laboratory Medicine, Gyeonsang National University Hospital, Gyeongsang National University College of Medicine, Jinju, South Korea
4. Department of Laboratory Medicine, National Health Insurance Service Ilsan Hospital, Goyang, South Korea
5. Department of Biomedical Laboratory Science, Yonsei University College of Health Sciences, Wonju, South Korea
6. Department of Internal Medicine, Yonsei University College of Medicine, Seoul, South Korea

Corresponding author: Kyungwon Lee, M.D., Ph.D.

Department of Laboratory Medicine and Research Institute of Bacterial Resistance

Yonsei University College of Medicine

50-1 Yonsei-ro, Seodaemun-gu

Seoul 03722, South Korea

Tel: +82-2-2228-2446

Fax: +82-2-313-0908

Email: leekcp@yuhs.ac

Alternate Corresponding author: Tae Il Kim, M.D., Ph.D.

Department of Internal Medicine

Yonsei University College of Medicine

50-1 Yonsei-ro, Seodaemun-gu

Seoul 03722, South Korea

Tel: +82-2-2228-1965

Fax: +82-2-313-0956

Email: taeilkim@yuhs.ac

Author M. Kim and author S-T Lee contributed equally to this manuscript.

Author K. Lee and author T.I. Kim contributed equally to this manuscript.

**Supplementary Table 1A. Detailed Characteristics of CRC patients**

| ID No.^a^ | Sex | Age (yrs) | Pathological Diagnosis | Carcinoma Location | Stage | Metastasis Organ |
| --- | --- | --- | --- | --- | --- | --- |
| 1 | F | 70 | Adenocarcinoma, moderately differentiated | Sigmoid colon | IIIB |  |
| 2 | M | 61 | Adenocarcinoma, moderately differentiated | Hepatic flexure | IV | Liver |
| 3 | F | 55 | Adenocarcinoma, moderately differentiated | Rectum | I |  |
| 4 | M | 71 | Adenocarcinoma, moderately differentiated | Sigmoid colon | IIA | Liver |
| 5 | M | 59 | Adenocarcinoma, moderately differentiated | Rectosigmoid junction | IV | Liver, Lung |
| 6 | M | 70 | Poorly differentiated carcinoma | Transverse colon | IIIB |  |
| 7 | M | 78 | Adenocarcinoma, moderately differentiated | Descending colon | IIA |  |
| 9 | M | 64 | Adenocarcinoma, moderately differentiated | Rectum | IIIB |  |
| 10 | F | 46 | Adenocarcinoma, moderately differentiated | Descending colon | I |  |
| 11 | M | 59 | Adenocarcinoma, well differentiated | Rectum | IIA |  |
| 12 | M | 67 | Adenocarcinoma, moderately differentiated | Rectum | IIA |  |
| 13 | F | 76 | Adenocarcinoma, moderately differentiated | Descending colon | IIA |  |
| 14 | M | 67 | Adenocarcinoma, well differentiated | Rectum | I |  |
| 15-1 | M | 70 | Adenocarcinoma, well differentiated | Rectum | IIIB |  |
| 15-2 | M | 70 | Adenocarcinoma, well differentiated | Hepatic flexure | IIIB |  |
| 16 | F | 84 | Adenocarcinoma with mostly signet ring cell features | Rectum | IVB |  |
| 17 | M | 73 | Adenocarcinoma, moderately differentiated | Sigmoid colon | IIA |  |
| 18 | F | 65 | Adenocarcinoma, well-differentiated | Ascending colon | IIIB |  |
| 19 | F | 61 | Adenocarcinoma, moderately differentiated | Sigmoid colon | IV | Liver, Lung |
| 20 | M | 70 | Adenocarcinoma, moderately differentiated | Sigmoid colon | IVB | Liver |
| 21 | M | 86 | Adenocarcinoma, moderately differentiated | Hepatic flexure | IV | Liver |
| 23 | F | 65 | Adenocarcinoma, moderately differentiated | Sigmoid colon | IV | Liver |
| 24 | M | 53 | Adenocarcinoma, moderately differentiated | Rectum | IIIB/C |  |
| 25 | M | 68 | Adenocarcinoma, moderately differentiated | Sigmoid colon | IV | Liver |
| 26 | M | 80 | Adenocarcinoma, moderately differentiated | Sigmoid colon | IIA |  |
| 27 | M | 49 | Adenocarcinoma, moderately differentiated | Rectum | IIIc | Liver |
| 28 | F | 65 | Adenocarcinoma, moderately differentiated | Hepatic flexure | IIA |  |
| 29 | F | 63 | Adenocarcinoma, moderately differentiated | Cecum | I |  |
| 30 | M | 59 | Adenocarcinoma, moderately differentiated | Sigmoid colon | IV | Liver |
| 31 | M | 86 | Adenocarcinoma, moderately differentiated | Ascending colon | IV | Liver, Lymph node |
| 32 | M | 77 | Adenocarcinoma, moderately differentiated | Ascending colon | III | Lymph node |
| 33 | M | 55 | Adenocarcinoma, moderately differentiated | Rectosigmoid junction | IV | Lymph node |
| 34 | F | 63 | Adenocarcinoma, moderately differentiated | Sigmoid colon | IIIc | Small intestine |
| 35 | F | 43 | Adenocarcinoma, moderately differentiated | Sigmoid colon | IV | Liver |
| 36 | F | 77 | Adenocarcinoma, moderately differentiated | Ascending colon | IIA |  |
| 37 | M | 45 | Adenocarcinoma, moderately differentiated | Sigmoid colon | IV | Liver, Lymph node |
| 38 | F | 75 | Adenocarcinoma with mucin pools | Ascending colon | IV | Bone, Lymph node |
| 39 | F | 69 | Adenocarcinoma, moderately differentiated | Rectum | IIIa |  |
| 40 | M | 56 | Adenocarcinoma, moderately differentiated | Rectum | IIIc | Liver |

^a^ID Cases No. 8 and No. 22 were excluded.

**Supplementary Table 1B. Detailed Characteristics of Controls**

| ID No. | Sex | Age (yrs) | Pathological Diagnosis | Tubular Adenoma Site | Number of polyps | Largest polyp size (mm) |
| --- | --- | --- | --- | --- | --- | --- |
| C1 | F | 47 | Tubular adenoma | Sigmoid colon | 1 | 3 |
| C2 | M | 45 | Tubular adenoma | Descending colon | 2 | 5 |
| C3 | M | 58 | Tubular adenoma | Ascending colon  Hepatic flexure | 2 | 7 |
| C4 | F | 54 | Tubular adenoma | Hepatic flexure | 1 | 5 |
| C5 | F | 61 | Tubular adenoma | Hepatic flexure | 2 | 3 |
| C6 | F | 58 | Normal | - | - | - |
| C7 | M | 59 | Normal | - | - | - |
| C8 | M | 65 | Tubular adenoma | Ascending colon | 2 | 4 |
| C9 | F | 66 | Normal | - | 1 | 7 |
| C10 | F | 69 | Tubular adenoma | Transverse colon | 1 | 3 |
| C11 | M | 62 | Tubular adenoma | Hepatic flexure | 5 | 5 |
| C12 | F | 69 | Tubular adenoma | Transverse colon | 1 | 4 |
| C13 | M | 73 | Tubular adenoma  Hyperplastic polyp | Cecum  Ascending colon  Transverse colon  Sigmoid colon | 7 | 7 |
| C14 | F | 67 | Normal | - | - | - |
| C15 | F | 63 | Tubular adenoma | Sigmoid colon | 2 | 7 |
| C16 | F | 70 | Tubular adenoma | Ascending colon  Descending colon | 2 | 4 |
| C17 | M | 62 | Normal | - | - | - |
| C18 | M | 72 | Normal | - | 2 | 3 |
| C19 | M | 71 | Normal | - | - | - |
| C20 | F | 67 | Normal | - | - | - |
| C21 | F | 68 | Hyperplastic polyp | - | 1 | 3 |

**Supplementary Figure 1.** Ct value of *F. nucleatum* in CT and AT of CRC patients in qPCR.

*F. nucleatum* was markedly enriched in carcinoma tissues (CT) compared to that in adjacent tissues (AT) of CRC patients and tissues (T) of controls (CT vs. T, CT vs. AT, and AT vs. T; Mann-Whitney U test; *P* < 0.0001, *P* < 0.0001, and *P* = 0.0078, respectively).

(A)


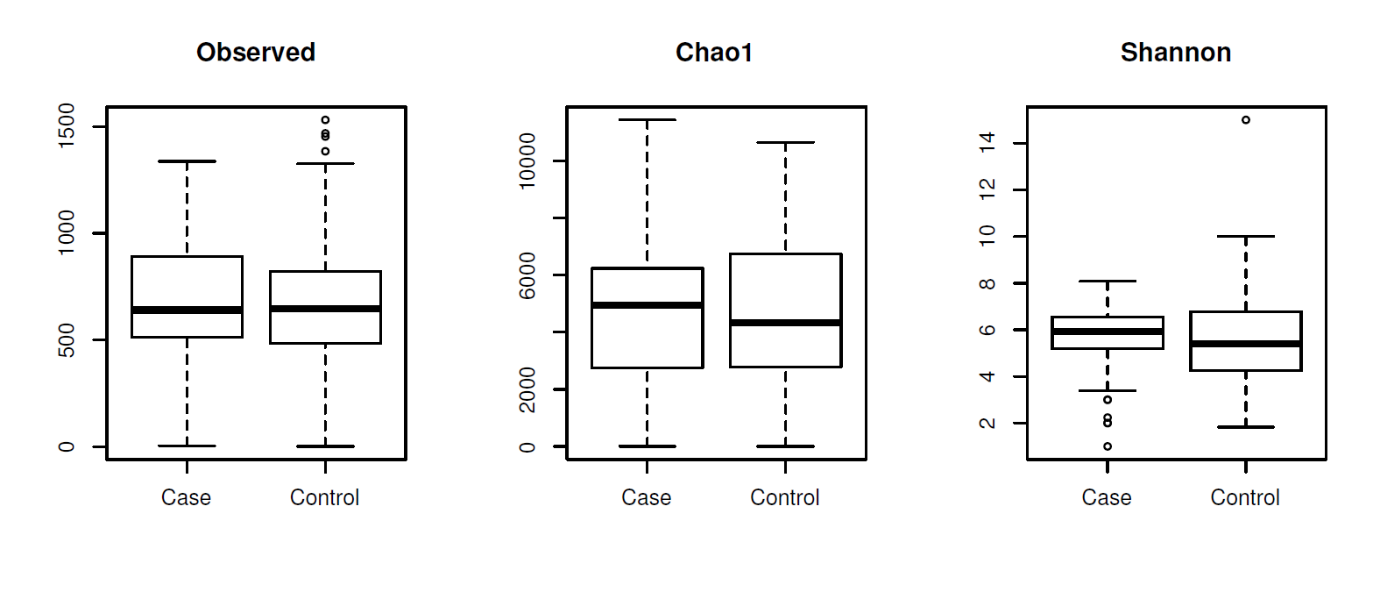


(B)


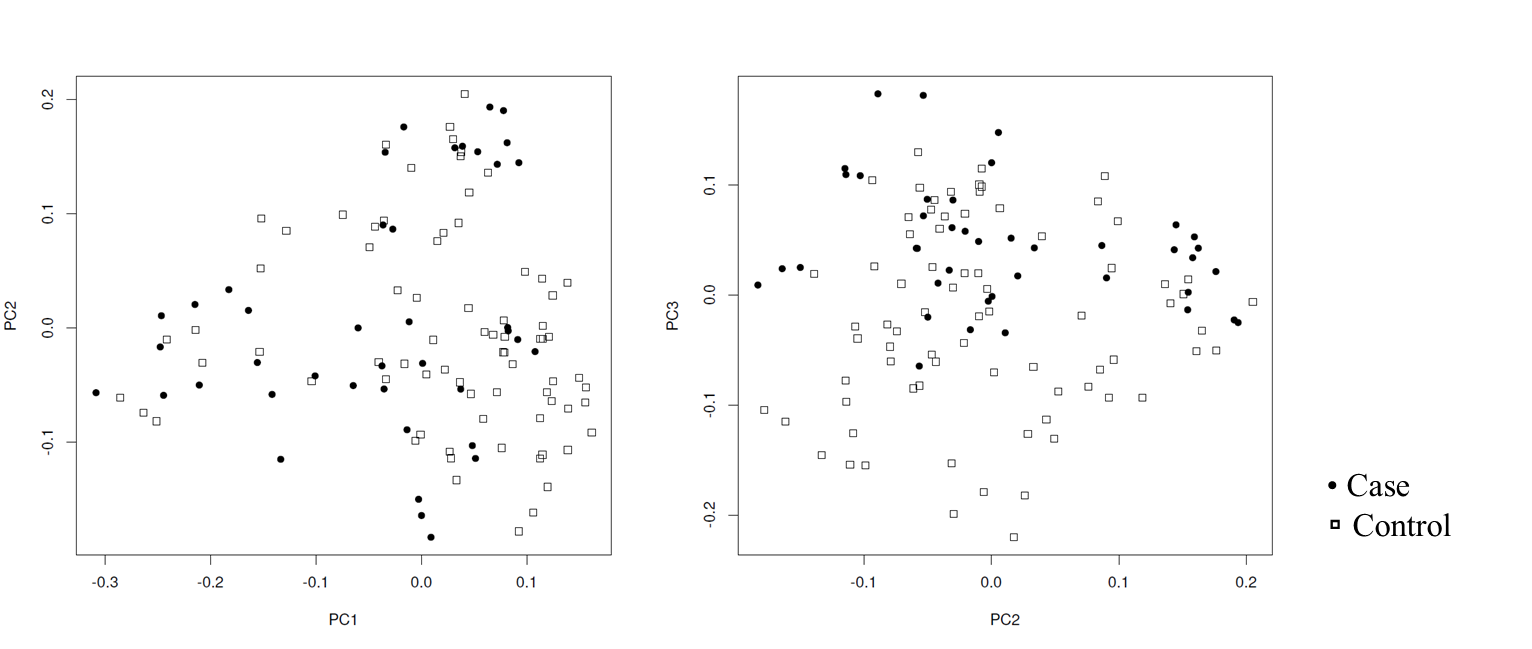


**Supplementary Figure 2**. α- (A) and β-diversities (B) of 16S rRNA analysis


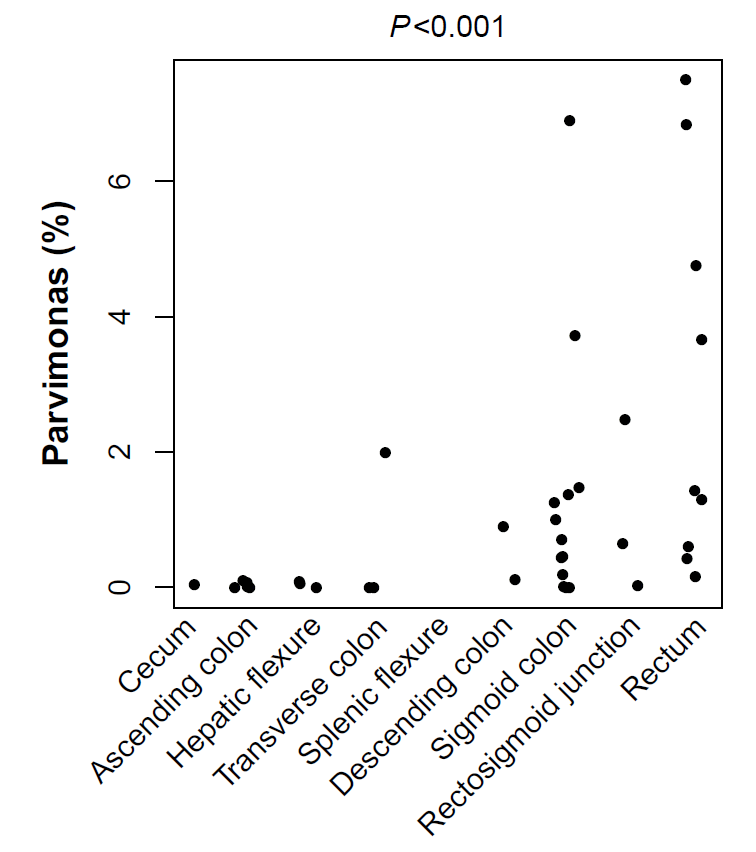


**Supplementary Figure 3.** Percentage of the *Parvimonas* OTU according to the location of the primary tumor
